# Endoscopic Management of Bleeding Ectopic Varices With Histoacryl

**DOI:** 10.1155/1999/35272

**Published:** 1999-04

**Authors:** Deepak K. Bhasin, B. C. Sharma, P. V. J. Sriram, G. Makharia, K. Singh

**Affiliations:** 1 Department of Gastroenterology Postgraduate Institute of Medical Education and Research Chandigarh 160012 India; 2 House No. 1041 Sector-24B Chandigarh 160 024 India

## Abstract

Bleeding from antral and duodenal varices is an uncommon feature in patients with portal hypertension. We report a patient with cirrhosis and portal vein thrombosis, who had a massive bleed from antral and duodenal varices. Bleeding was controlled with endoscopic injection of varices using histoacryl. Endoscopic treatment and the relatively uncommon occurrence of antral and duodenal varices are highlighted.


*HPB Surgery*, 1999, Vol. 11, pp. 171–173
Reprints available directly from the publisher
Photocopying permitted by license only

© 1999 OPA (Overseas Publishers Association) N.V.
Published by license under
the Harwood Academic Publishers imprint,
part of The Gordon and Breach Publishing Group.
Printed in Malaysia.

### Endoscopic Management of Bleeding Ectopic Varices with Histoacryl

DEEPAK K. BHASIN*, B. C. SHARMA, P. V. J. SRIRAM, G. MAKHARIA and K. SINGH

*Department of Gastroenterology, Postgraduate Institute of Medical Education and Research, Chandigarh-160012, India*

*(Received 14 June 1997; In final form 14 December 1997)*

**Bleeding from antral and duodenal varices is an uncommon feature in patients with portal hypertension. We report a patient with cirrhosis and portal vein thrombosis, who had a massive bleed from antral and duodenal varices. Bleeding was controlled with endoscopic injection of varices using histoacryl. Endoscopic treatment and the relatively uncommon occurrence of antral and duodenal varices are highlighted.**

*Keywords:* Gastric varices, duodenal varices, antral varices, *n*-butyl 1-2-cyanoacrylate, glue, cirrhosis, portal vein thrombosis, sclerotherapy

### INTRODUCTION

Varices can occur all along the gastrointestinal tract in patients with portal hypertension [1, 2]. Antral and duodenal varices are relatively uncommon and bleed rarely. However bleeding from antral and duodenal varices is usually massive and often fatal [3–5]. Till now there are few case reports on endoscopic hemostasis of bleeding duodenal varices. Also there is paucity of reports on endoscopic injection of antral varices with histoacryl. We report a patient having bleeding from fundal, antral and duodenal varices, which could be successfully controlled with endoscopic injection using histoacryl.

### CASE REPORT

A 50 year old lady presented with recurrent hematemesis and melena for nine months. On admission she had severe anemia, pedal edema, and ascites Viral (HBsAg and anti HCV antibodies [second generation ELISA]) and autoimmune markers (antinuclear factor and antismooth muscle antibodies) were negative. On ultrasound the liver was shrunken with irregular border and altered echotexture. Portal vein revealed a large echogenic thrombus. Upper gastrointestinal endoscopy revealed grade III esophageal varices and large varices in the fundus of stomach, antrum and the second part of duodenum. Esophageal varices were eradicated after five sessions of sclerotherapy. However after 3 months of sclerotherapy the patient again developed massive hematemesis. On urgent endoscopy active bleeding from

*Address for correspondence: House No. 1041, Sector-24B, Chandigarh - 160 024, India.

fundal and duodenal varices was demonstrated, and an antral varix showed hyperemia. Fundal, antral and duodenal varices were injected with 0.5 ml of histoacryl and 0.5 ml of lipiodol mixture at each site. Following injection bleeding stopped and there were no post-procedure complications. Patient did not require repeat injection. Over follow up of one year there is no rebleeding and all the three ectopic varices are markedly reduced in size on endoscopy.

### DISCUSSION

Ectopic varices are defined as those arising in sites other than the esophagus and cardia of the stomach [2]. They may follow injection sclerotherapy and obliteration of esophageal varices or develop spontaneously [2]. Ectopic varices are sites of bleeding in 1–5% of patients with intrahepatic portal hypertension and 20–30% of those with extrahepatic portal hypertension [1, 2, 6].

Approximately 40% of patients with portal hypertension have paraduodenal varices on angiography [7]. Endoscopic evidence of duodenal varices is uncommon, which is probably due to deeper location in the intestinal wall. Duodenal varices being deeper have a lower risk of bleeding compared to submucosal esophageal varices in the distal esophagus [3]. Endoscopically visible submucosal duodenal varices with a tendency to bleed generally result from previous sclerotherapy of esophageal varices [8]. Our patient had duodenal varices even before initiating sclerotherapy of esophageal varices. Injection sclerotherapy of duodenal varices is technically difficult since compression on the bleeding site caused by injection, with shaft of endoscope is not possible [9]. Different sclerosing agents like polidocanol and ethanolamine oleate have been used [10, 11]. There are only a few reports of histoacryl injection in bleeding duodenal varix [9, 12]. Since histoacryl is more potent than other agents, only 1–2 sessions are required to eradicate duodenal varices [9, 12]. In majority of cases antral varices also appear after sclerotherapy and obliteration of esophageal varices [13]. In our patient antral varix could be seen before starting the patient on sclerotherapy programme for esophageal varices.

Ectopic varices are more common in extrahepatic portal venous obstruction [1]. The coexistence of portal vein thrombosis with cirrhosis in our patient may be responsible for development of ectopic varices at multiple sites. In older series, the reported frequency of portal vein thrombosis in patients with cirrhosis is 10–11% [14]. However in large prospective series by Okuda *et al.*, frequency of portal vein thrombosis was found to be 0.54% on angiographic evaluation of portal vein in patients with cirrhosis [15]. The coexisting portal vein thrombosis with cirrhosis is another rare feature in our patient. However a high frequency of portal vein thrombosis is reported in patients with cirrhosis developing hepatocellular carcinoma, which was excluded in our patient.

Endoscopic injection using histoacryl is an effective mode of treatment for bleeding ectopic varices.

### *References*

[1] Lebrec, D. and Benhamou, J. P. (1985). Ectopic varices in portal hypertension, *Clin. Gastroenterol.*, **14**, 105–21.
[2] Heaton, N. D., Khawaja, H. and Howard, E. R. (1991). Bleeding duodenal varices, *Br. J. Surg.*, **78**, 1450–1.
[3] Nardone, G. and Budillon, G. (1991). Treatment of duodenal varices by endoscopic sclerotherapy, *Gastrointest Endosc.*, **37**, 407–8.
[4] Itzchak, Y. and Glickman, M. G. (1976). Duodenal varices in extrahepatic portal obstruction, *Radiology*, **124**, 619–24.
[5] Kunert, H. and Ottenjann, R. (1976). Endoscopy in bleeding duodenal varices, *Endoscopy*, **8**, 99–101.
[6] Kinkhabwala, M., Mousavi, A., Iyer, S. *et al.* (1977). Bleeding ileal varicosity demonstrated by transhepatic portography, *Am. J. Radiol.*, **129**, 514–6.
[7] Stephan, G. and Miething, R. (1968). Rontgen-diagnostic varicoser Duodenal veranderungen bei portaler hypertension, *Der Radiologie*, **3**, 90–5.
[8] Sauerbruch, T., Weinzierl, M., Dietrich, H. P. *et al.* (1982). Sclerotherapy of a bleeding duodenal varix, *Endoscopy*, **14**, 187–9.

[9] Sung, J. Y., Chung, S. C. S., Leung, H. T. *et al.* (1993). Duodenal varices in hepatocellular carcinoma, *Endoscopy*, **25**, 194–6.
[10] Gertsch, P. H. and Blumgart, L. H. (1988). Cure of a bleeding duodenal varix by sclerotherapy, *Br. J. Surg.*, **75**, 717.
[11] Wu Shyond, C., Chen, C. M. and Chang, K. Y. (1995). Endoscopic injection sclerotherapy of bleeding duodenal varices, *J. Gastroenterol. and Hepatol.*, **10**, 481–3.
[12] Labenz, J. and Borsch, G. (1993). Successful endoscopic hemostasis of duodenal variceal bleeding with histoacryl, *Endoscopy*, **25**, 194.
[13] Sarin, S. K., Lahoti, D., Saxena, S. P. *et al.* (1992). Prevalence, classificaton and natural history of gastric varices. A long term follow up study in 568 portal hypertension patients, *Hepatology*, **16**, 1343–9.
[14] Hunt, A. H. and Whittard, B. R. (1954). Thrombosis of portal vein in cirrhosis hepatis, *Lancet*, **1**, 281.
[15] Okuda, K., Ohnishi, K., Kimura, K. *et al.* (1985). Incidence of portal vein thrombosis in liver cirrhosis. An angiographic study of 708 patients, *Gastroenterology*, **89**, 279–86.

### COMMENTARY

This is a case history of successful endoscopic management of bleeding ectopic varices in antrum and duodenum with injection of histoacryl.

The appearance of varices in duodenum and antrum is an uncommon complication to portal hypertension and is most often seen in patients with an extra hepatic block in the portal vein as the author states. The patient apparently also has had oesophageal varices treated with injection of absolute alcohol which is a technique inferior to other sclerosants (Attam Kurri, S.P., Bhargava, D.K. and Sharma, M.P. (1988) Endoscopic sclerotherapy for oesophageal varices: a prospective, randomized trial of absolute alcohol versus polidocanal, Indian J. Gastroenterol, 7, 87–89.

Injection of ordinary sclerosants in duodenum may have deleterious side effects and therefore other occlusive materials acting locally in the blood vessels should be used and the use of histoacryl is one such agent. A similar approach has been successfully used in management of fundic varices.

Prof. B W Jeppsson

Department of Surgery

Malmö University Hospital

Malmö

S-205 02

Sweden

